# Supplementary figures and images for: Determination of oligomeric states of proteins via dual-color colocalization with single molecule localization microscopy
Source: eLife. 2022 Oct 7;11:e76631. doi: 10.7554/eLife.76631 (PMC9584609; doi:10.7554/eLife.76631)

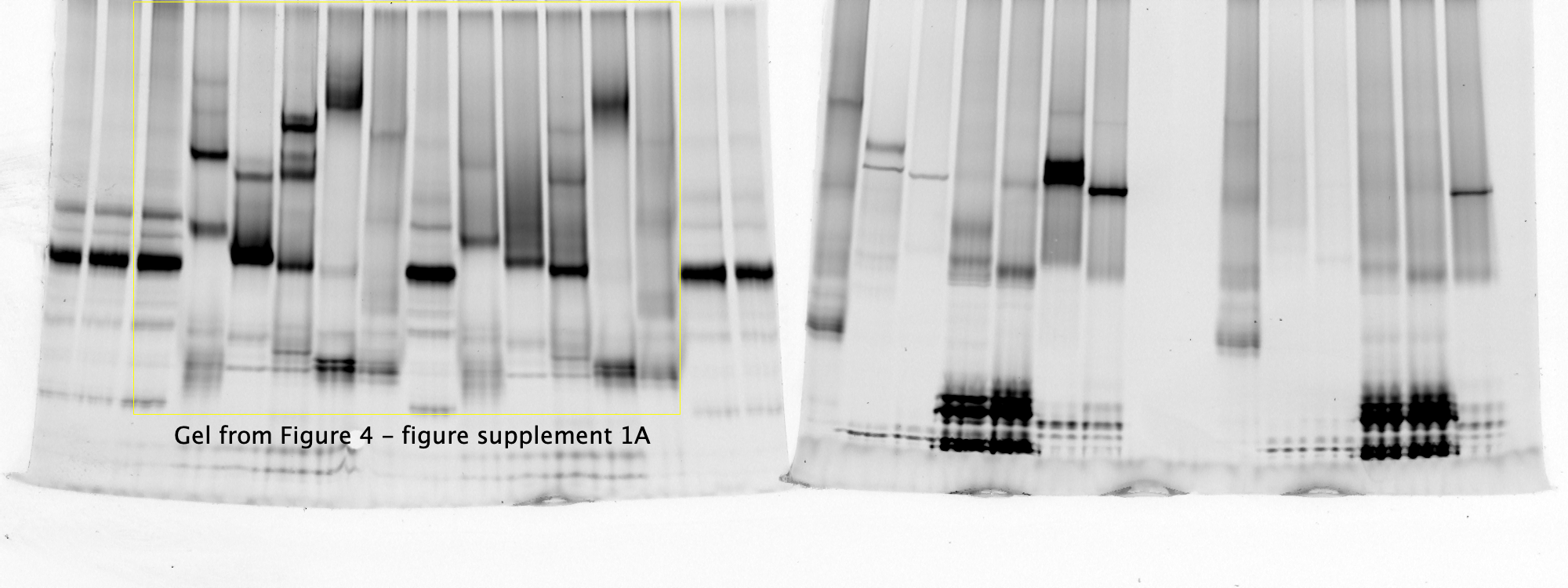

Supplement: Figure 4—figure supplement 2—source data 1. [file elife-76631-fig4-figsupp2-data1.zip › Gel_A_marked.tif]

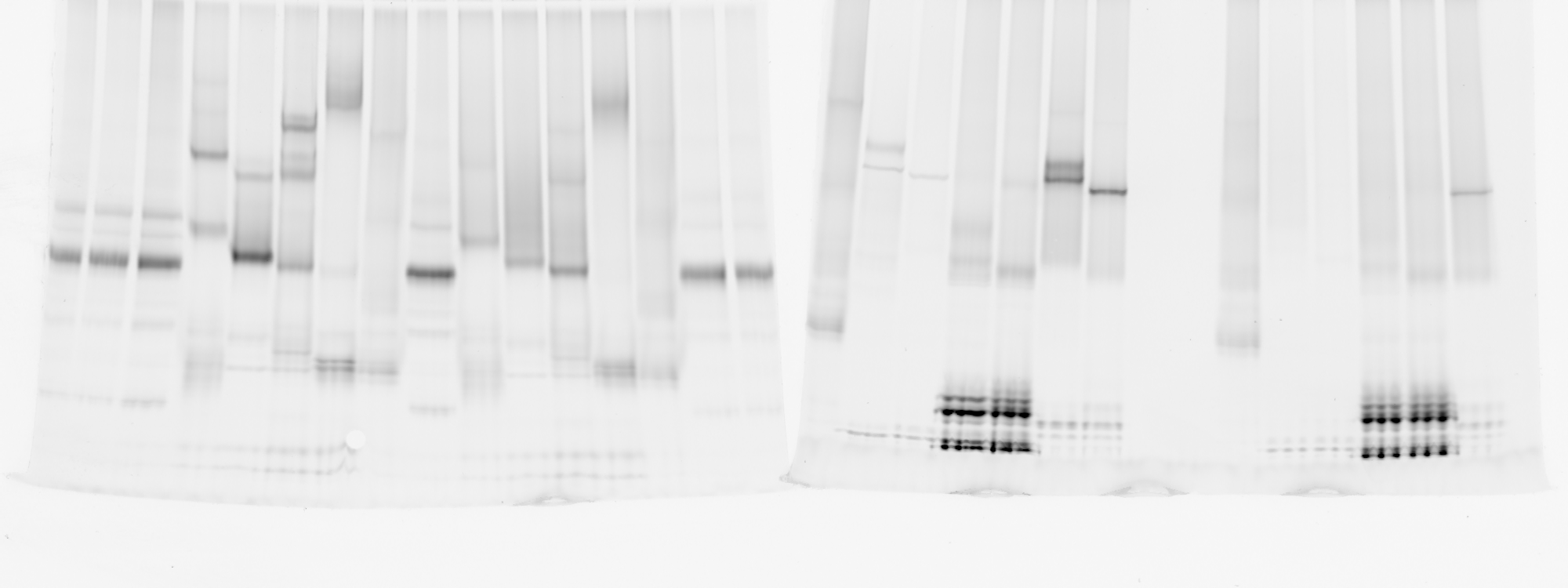

Supplement: Figure 4—figure supplement 2—source data 1. [file elife-76631-fig4-figsupp2-data1.zip › Gel_A.tif]

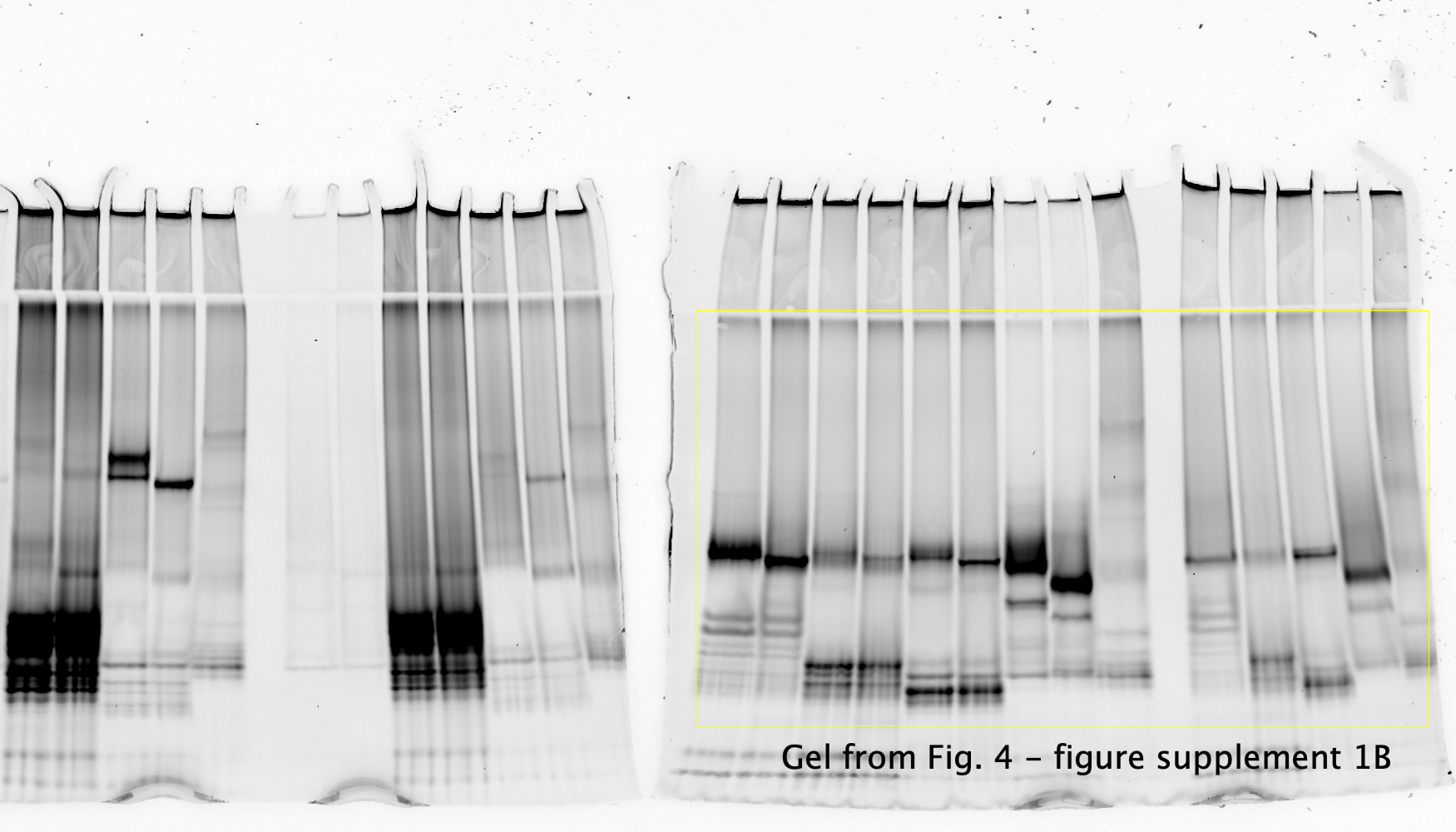

Supplement: Figure 4—figure supplement 2—source data 1. [file elife-76631-fig4-figsupp2-data1.zip › Gel_B_marked.tif]

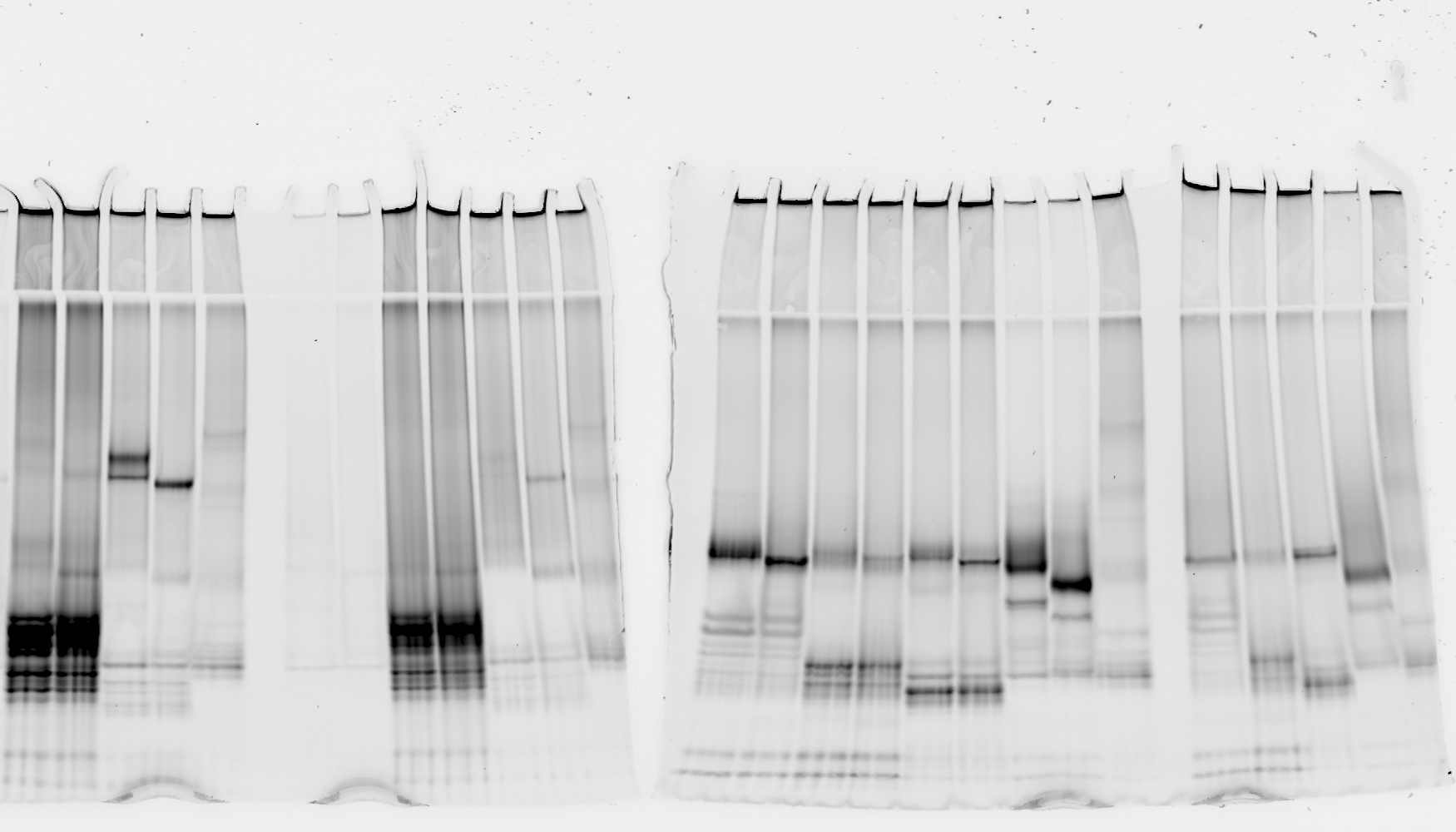

Supplement: Figure 4—figure supplement 2—source data 1. [file elife-76631-fig4-figsupp2-data1.zip › Gel_B.tif]

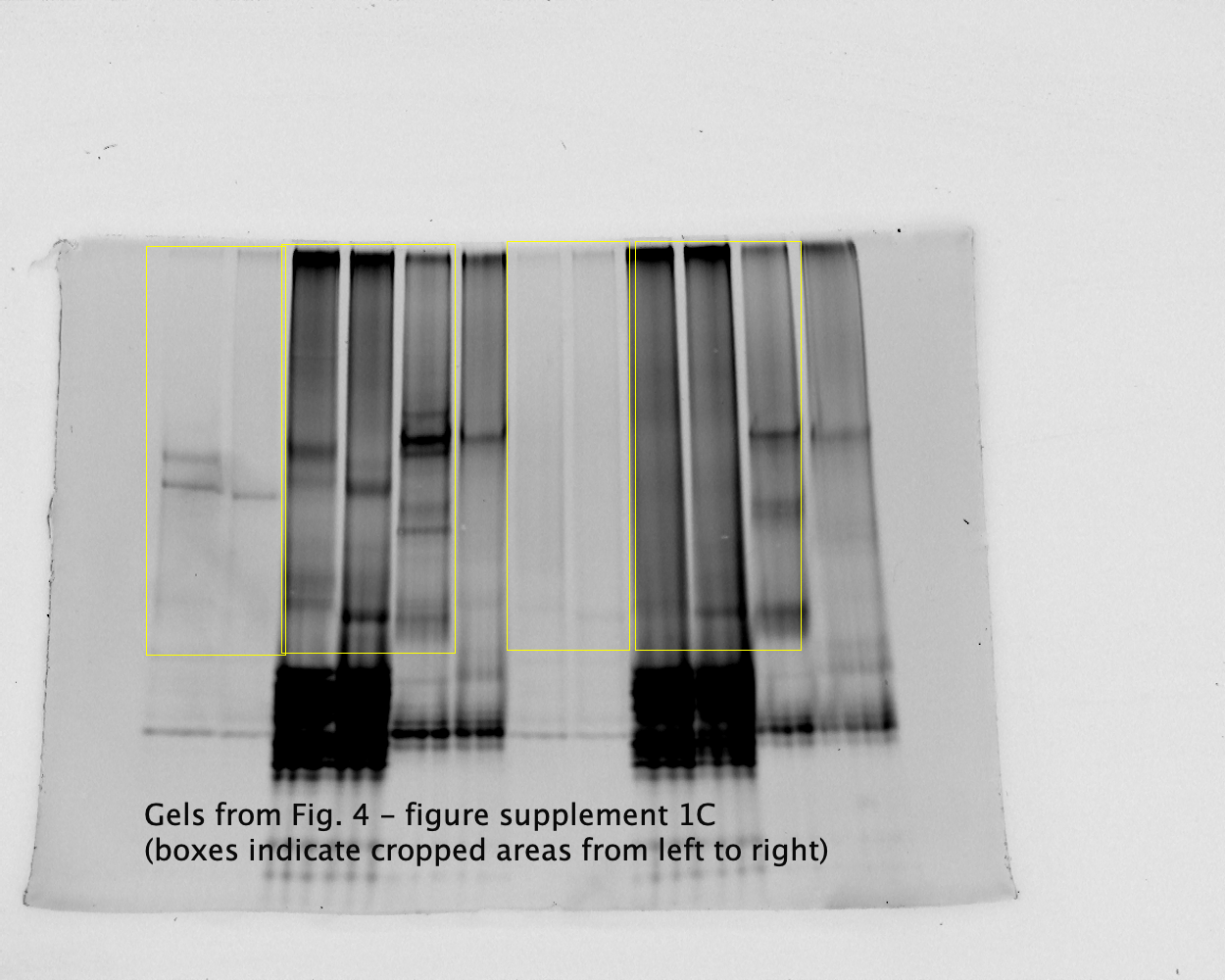

Supplement: Figure 4—figure supplement 2—source data 1. [file elife-76631-fig4-figsupp2-data1.zip › Gel_C_marked.tif]

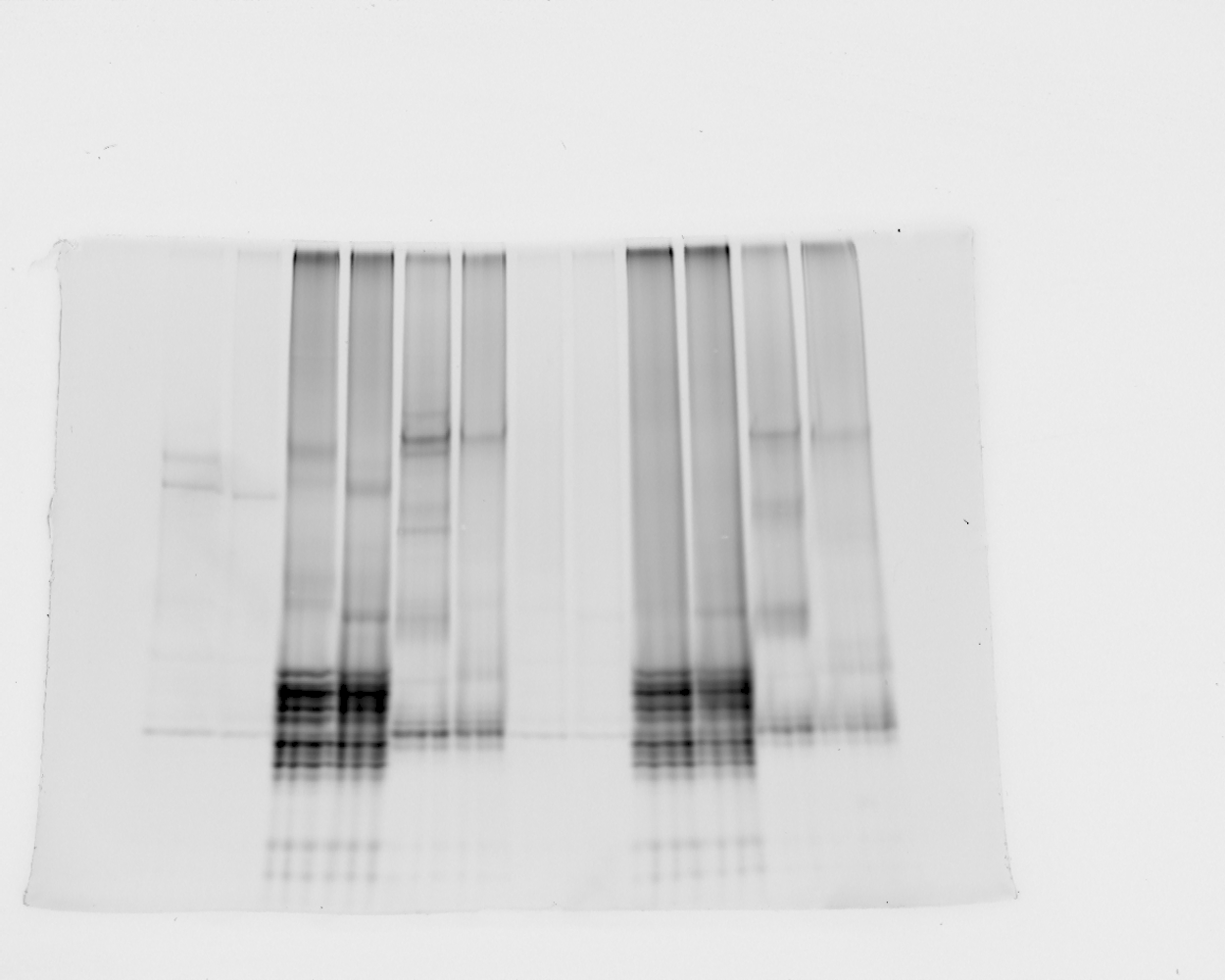

Supplement: Figure 4—figure supplement 2—source data 1. [file elife-76631-fig4-figsupp2-data1.zip › Gel_C.tif]
